# Effect of epiphytic microflora after aerobic enrichment and reconstitution on fermentation quality and microbial community of corn stalk silage and *Pennisetum sinese* silage

**DOI:** 10.3389/fmicb.2022.1078408

**Published:** 2022-12-01

**Authors:** Yixiao Xie, Ermei Du, Yudie Yao, Wanqing Wang, Xiaokang Huang, Hong Sun, Yulong Zheng, Qiming Cheng, Chunmei Wang, Chao Chen, Ping Li

**Affiliations:** ^1^College of Animal Science, Guizhou University, Guiyang, Guizhou, China; ^2^Key Laboratory of Animal Genetics, Breeding and Reproduction in the Plateau Mountainous Region, Ministry of Education, Guizhou University, Guiyang, Guizhou, China

**Keywords:** anaerobic fermentation, corn stalk, *Pennisetum sinese*, microbial community, pre-incubated juice

## Abstract

**Introduction:**

The objective of this study was to evaluate the effects of enrichment and reconstitution of the forage epiphytic microflora on the fermentation quality, chemical composition, and bacterial community composition of corn stalk and *Pennisetum sinese* silages.

**Methods:**

The forage juice of fresh corn stalk and *P. sinese* were collected, diluted by gradient (10^–1^ to 10^–5^), and aerobically incubated to enrich and reconstitute the epiphytic microflora. Fresh corn stalk and *P. sinese* were ensiled for 3, 15, and 45 days after inoculation with either the original (D0) pre-incubated juices, or 10^–1^ (D1), 10^–3^ (D3), or 10^–5^ (D5) diluted and pre-incubated juices.

**Results:**

The lowest pH was found in the D3 treatment of the corn stalk silage. In *P. sinese* silage, the hemicellulose content of D3 and D5 treatments was 9.50 and 11.81% lower than that of D0 treatment (*P* < 0.05). In corn stalk silage, the neutral detergent fiber content was significantly lower in the D3 treatment than in the other treatments (*P* < 0.05). Both corn stalk and *P. sinese* silages exhibited a high abundance of *Enterobacter* during ensiling, resulting in high levels of acetic acid.

**Conclusion:**

Although the dilution and enrichment of the epiphytic microflora did not lead to full lactic acid fermentation, these pre-treatments were found to alter the microbial metabolites and chemical composition of the silage. These results provide a new perspective on the production of pre-fermented silage inoculant.

## Introduction

In China, corn stalk and *Pennisetum sinese* are the most high-yielding sources of forage material. Anaerobic fermentation of forage material, or ensiling, results in the conversion of soluble carbohydrates into a variety of end products, allowing the nutrition in the biomass to be preserved for later use as a livestock feed (Kung et al., 2018). In addition, ensiling increases microbial activity and produces an acidic environment through the production of organic acids, which disrupts the structure of lignocellulose and creates a more accessible area for microbial activity and enzymatic hydrolysis, thus improving the biodigestibility of lignocellulose (Sun et al., 2021). Ensiling is suitable for long-term preservation of high-moisture, perishable fresh crops or biomass. Compared with the fresh crop, the silage is a production with a high recovery of dry matter, energy, and highly digestible nutrients (Kung et al., 2018). Therefore, ensiling of corn stalk and *P. sinese* with high moisture and sugar content is not only a good way to preserve feed, but is also a feasible pretreatment technology to improve the biodigestibility of biomass for the production of bioenergy.

Lactic acid bacteria (LAB), which are typically used as inoculants in silage processing, utilize the soluble carbohydrates in the forage to produce lactic acid, resulting in acidification of the silage (Muck et al., 2018). The LAB isolated from different crops and environments can have diverse effects on silage fermentation, although studies have shown that the most effective inoculant may come from the crop itself (Weinberg and Muck, 1996; Yan et al., 2019). Therefore, to harness the power of naturally occurring epiphytic LAB, a liquid forage extract (“forage juice”) can be produced and subsequently aerobically fermented to enhance LAB abundance and produce an effective silage inoculant comparable to commercial inoculants. Inoculating low sugar, high buffering capacity legume forage with pre-fermented, LAB-enriched forage juice has been shown to improve aerobic stability fermentation quality and reduce protein degradation in the silage (Wang et al., 2009; Denek et al., 2011; Mu et al., 2022).

However, in a recent study, we used an ultraviolet reduction approach to enrich the epiphytic microflora, including both LAB and enterobacteria, of oat silage and observed improved fermentation quality and enhanced methane emissions (Xie et al., 2022). The enrichment of epiphytic microflora by using aerobically incubated forage juice as an inoculant has not been investigated. Based on previous results, it can be expected that the inoculation may also contribute positively to the subsequent anaerobic fermentation. In addition, research has shown that soil microbial diversity can be reconstituted by diluting microflora with a gradient and then re-enriching it (Philippot et al., 2013). The gradient dilution will also be applied in this study to reschedule the enriched microflora. It can be hypothesized the rescheduling of the epiphytic microbial diversity could further alter the initiation of anaerobic fermentation and thus exert an unknown influence on the ensiling process.

The fermentation process of silages is divided into different phases: initial aerobic phase, fermentation phase, and stable phase (Soundharrajan et al., 2021). Researchers generally analyze the bacterial community composition at key time points in the different phases of fermentation (e.g., 5, 15, and 45 days) with a view to explain the changes in the fermentation quality and chemical composition of silage (Mu et al., 2022). The objective of this study was to evaluate the effects of enrichment and reconstitution of epiphytic forage microflora on the chemical composition, dynamics of fermentation quality and bacterial community composition of corn stalk and *P. sinese* silages.

## Materials and methods

### Materials and preparation of pre-incubated forage juice

Both corn stalk (ear-removed, “Jinyu908”) and *P. sinese* were manually harvested from an experimental field at Guizhou University in Guanling County, Guizhou Province, China. The corn stalk was harvested at the dough stage, leaving a 30-cm stubble. The *P. sinese* was harvested at physiologic maturity stage, leaving a 15-cm stubble. The corn stalk demonstrated a dry matter (DM) content of 22.65% fresh matter, a water-soluble carbohydrate (WSC) content of 9.57% DM, a crude protein (CP) content of 9.42% DM, a neutral detergent fiber (NDF) content of 58.50% DM, an acid detergent fiber (ADF) content of 38.19% DM, and a hemicellulose content of 20.31% DM. The *P. sinese* demonstrated a DM content of 24.88% fresh matter, a WSC content of 5.36% DM, a CP content of 4.20% DM, a NDF content of 76.26% DM, an ADF content of 52.83% DM, and a hemicellulose content of 23.43% DM.

To make the forage juice, 150 g of fresh corn stalk or *P. sinese* was chopped, mixed with 150 mL of sterilized water, and homogenized in a laboratory juicer. A 12-mL subsample of the original (10^0^, D0) forage juice was blended with 108 mL of sterilized water and serially diluted to 10^–1^ (D1), 10^–3^ (D3), and 10^–5^ (D5). To each 100-mL of diluted sample (including D0), 2 g of sucrose was added and mixed. The resulting solutions were transferred into 250-mL sterile triangular flasks, which were sealed with a breathable membrane. The triangular flasks were placed in a constant-temperature incubator and incubated at 37°C for 24 h. The pre-incubated forage juices were used to inoculate the forages prior to ensiling.

### Silage preparation

Fresh corn stalk or *P. sinese* was chopped to approximately 2–3 cm and randomly divided into 36 individual 150-g samples per species. The 36 samples (per species) were randomly divided into four treatments and inoculated with either D0, D1, D3, or D5 pre-incubated forage juices at a rate of 7.5 mL per sample, respectively. Silage was prepared by vacuum sealing the inoculated plant material into polyethylene bags (25 × 30 cm). Additional 0-day samples were inoculated without sealing. The selection of time points for the determination of parameters was based on Mu et al. (2022). Specifically, we modified the time points to day 3, day 15, and day 45 due to the more rapid initiation of fermentation in grasses. After 3, 15, and 45 days of ensiling, three bags from each treatment were unpacked, sampled, and analyzed.

### Physical and chemical analysis

After unpacking and thoroughly blending the contents of each bag, 20 g samples were collected, mixed with 180 mL of distilled water, and homogenized for 60 s in a laboratory juicer. Each sample was subsequently filtered through four layers of cheesecloth and centrifuged at 6,500 rpm at 4°C for 15 min. The supernatant was collected to analyze the pH, ammonia nitrogen (NH_3_-N), and organic acid content. The pH was measured using a pH meter. The NH_3_-N content was analyzed according to the sodium hypochlorite and phenol method (Broderick and Kang, 1980). The lactic, acetic, propionic, and butyric acid contents were determined by high-performance liquid chromatography (HPLC, Shimadzu, Japan) according to Xie et al. (2021). Approximately 100 g of each 45-day silage sample was dried in a forced-draft oven at 65°C for 48 h to determine the DM content. Each dried 60-day silage sample was ground through a 0.20-mm sieve and then used to determine the WSC, CP, NDF, and ADF contents. The WSC content was determined by the method of Arthur Thomas (1977). The CP content was determined by the method AOAC (1990). The NDF and ADF contents were determined according to the procedure of Van Soest et al. (1991). The hemicellulose contents were estimated as the NDF values minus the ADF values.

### Bacterial community analysis

The bacterial community compositions of both forage and silage were profiled according to the method of Xie et al. (2022). Briefly, 10 g of either fresh forage or silage sample was blended with 40 mL of saline solution (NaCl, 8.5 g kg^–1^) and shaken for 120 min at 120 rpm. The homogenized liquor was filtered through two layers of cheesecloth and centrifuged at 10,000 rpm at 4°C for 15 min. The supernatant was discarded and the pellet was used to extract DNA. The primer pair 338F (5′-ACTCCTACGGGAGGCAGCAG-3′) and 806R (5′-GGACTACHVGGGTWTCTAAT-3′) was used to amplify the 16S rRNA gene. The PCR products were purified using a Tiangen Gel Extraction Kit (Tiangen, Beijing, China). The purified DNA samples were sent to Magigene Company (Shenzhen, China) for 16S rRNA gene amplicon sequencing using paired-end (PE) sequencing with an Illumina HiSeq PE2500 platform. Based on the operational taxonomic unit (OTU) results, alpha diversity analyses were calculated using Mothur (version 1.30.1) (Schloss et al., 2011).

### Statistical analyses

The data were subjected to one-way and two-way analyses of variance (ANOVA) with the fixed effects of dilution gradient and storage period using SPSS version 19.0 for Windows (SPSS Inc., Chicago, IL, USA). Duncan’s multiple range test was carried out to determine the statistically significant differences among the means across dilution gradients and storage periods. For all analyses, statistical significance was declared at *P* < 0.05.

## Results

### Fermentative characteristics of silages during ensiling

Alterations in fermentation characteristics during anaerobic fermentation are shown in Table 1. No significant interaction was observed between dilution gradients and storage periods in the fermentative characteristics of the silages. Because neither propionic nor butyric acid were detected in either the corn stalk or *P. sinese* silage, these acids were not included in the table. Of the corn stalk silage, the D3 treatment exhibited the lowest pH. Of the *P. sinese* silage, both the D3 and D5 treatments exhibited significantly lower pH than the D1 or D0 treatments (*P* < 0.05). The pH of each treatment decreased significantly after 15 days of fermentation (*P* < 0.05). Additionally, the lactic and acetic acid contents of the corn stalk silage on day 3 was significantly lower than at later time periods.

**TABLE 1:** Fermentative characteristics of corn stalk and *Pennisetum sinese* silages during ensiling.

Forage	Attribute	Dilution	Storage period (days)			SEM	*P-*value		
			3	15	45		D	S	D × S
Corn stalk	pH	D0	4.62	4.57	4.47	0.04	0.040	0.001	0.279
		D1	5.03	4.47	4.69				
		D3	4.80	4.19	4.19				
		D5	4.69	4.52	4.39				
	Lactic acid (% DM)	D0	3.93	5.08	6.51	0.36	0.534	0.033	0.905
		D1	1.85	5.19	5.13				
		D3	4.50	5.76	5.41				
		D5	2.58	5.44	4.50				
	Acetic acid (% DM)	D0	5.34	6.28	8.37	0.54	0.698	0.042	0.830
		D1	2.37	6.62	8.74				
		D3	4.06	6.51	5.42				
		D5	3.00	6.27	5.56				
	NH_3_-N (% TN)	D0	3.96	2.73	2.86	0.14	0.065	0.410	0.206
		D1	3.33	2.80	3.92				
		D3	4.02	3.69	4.37				
		D5	4.29	4.55	3.36				
*Pennisetum sinese*	pH	D0	5.71	5.22	5.09	0.04	<0.001	<0.001	0.557
		D1	5.36	4.93	5.00				
		D3	5.12	4.64	4.77				
		D5	4.93	4.43	4.87				
	Lactic acid (% DM)	D0	2.67	2.41	2.27	0.17	0.867	0.403	0.894
		D1	2.55	3.51	2.19				
		D3	2.71	2.57	2.28				
		D5	2.10	2.75	2.27				
	Acetic acid (% DM)	D0	4.58	3.40	3.65	0.24	0.463	0.935	0.760
		D1	3.52	4.44	3.70				
		D3	3.54	2.95	3.44				
		D5	2.28	2.86	3.71				
	NH_3_-N (% TN)	D0	2.99	2.75	3.67	0.20	0.096	0.251	0.867
		D1	4.67	3.94	5.13				
		D3	3.87	4.49	4.27				
		D5	3.32	4.08	4.86				

NH_3_-N, ammonia nitrogen; DM, dry matter; TN, total nitrogen; D0, undiluted; D1, 10^–1^ dilution; D3, 10^–3^ dilution; D5, 10^–5^ dilution; D, dilution gradient; S, storage period; D × S, interaction between dilution gradient and storage period; SEM, standard error of the mean.

### Chemical composition of silages after ensiling

The chemical compositions of silages after 45 days of ensiling are shown in Table 2. In both the corn stalk and *P. sinese* silages, the highest residual WSC was found in the D1 treatment (*P* < 0.05). In both the corn stalk and *P. sinese* silages, there were no significant differences in the DM, CP, or ADF content between the treatment (*P* > 0.05). In corn stalk silage, the NDF content of the D3 treatment decreased by 2.88% compared to the D0 treatment, which was significantly lower than the other three treatments (*P* < 0.05). Both the D3 and D5 treatments exhibited lower hemicellulose compared to the D1 and D0 treatments. Specifically, the hemicellulose content of the D3 and D5 treatments was 9.50 and 11.81% lower than that of D0 treatment, respectively, which was significantly lower than that of D1 and D0 treatments (*P* < 0.05).

**TABLE 2:** Chemical composition of corn stalk and *Pennisetum sinese* silages after 45 days of ensiling.

Forage	Attribute	D0	D1	D3	D5	SEM	*P-*value
Corn stalk	Dry matter (% FM)	20.13	20.24	21.11	20.38	0.32	0.757
	Water-soluble carbohydrate (% DM)	6.39^a^	4.50^c^	4.97^bc^	5.42^b^	0.22	<0.001
	Crude protein (% DM)	10.20	11.25	10.10	9.76	0.31	0.388
	Neutral detergent fiber (% DM)	58.27^a^	58.58^a^	56.59^b^	58.43^a^	0.31	0.039
	Acidic detergent fiber (% DM)	38.70	39.82	39.78	39.29	0.23	0.308
	Hemicellulose (% DM)	19.58	18.75	16.81	19.14	0.43	0.081
*Pennisetum sinese*	Dry matter (% FM)	24.51	23.30	23.98	20.02	0.71	0.080
	Water-soluble carbohydrate (% DM)	2.75^b^	1.94^c^	2.85^b^	3.59^a^	0.18	<0.001
	Crude protein (% DM)	4.46	4.28	5.28	4.65	0.22	0.466
	Neutral detergent fiber (% DM)	75.05	77.17	72.41	74.01	0.69	0.069
	Acidic detergent fiber (% DM)	52.62	54.68	52.11	54.23	0.62	0.437
	Hemicellulose (% DM)	22.43^a^	22.50^a^	20.30^b^	19.78^b^	0.44	0.020

Means within the same row (a–c) with different superscripts differ significantly from each other (*P* < 0.05).

FM, fresh matter; DM, dry matter; D0, undiluted; D1, 10^–1^ dilution; D3, 10^–3^ dilution; D5, 10^–5^ dilution; D, dilution gradient; SEM, standard error of the mean.

### Bacterial compositions of silages during ensiling

In the *P. sinese* silage, significant interactions between the dilution gradients and storage periods were observed for both the ACE and Chao1 indices (*P* < 0.05) (Table 3). Because the Goods-coverage of all samples was greater than 0.999, the Goods-coverage was not included in the table, although these results indicate that all samples had adequate coverage. In corn stalk silage, the ACE index was higher at day 15 than at day 45 (*P* < 0.05), implying that community richness declined over time. A similar trend was observed in the D0 and D3 treatments of *P. sinese* silage. In corn stalk silage, the Simpson index of the D0 treatment was significantly lower than that of the other treatments (*P* < 0.05), while the Shannon index was significantly higher than that of the other treatments (*P* < 0.05), implying that the D0 treatment had the highest bacterial community diversity (*P* < 0.05).

**TABLE 3:** Alpha diversity indices of corn stalk and *Pennisetum sinese* silages during ensiling.

Forage	Index	Dilution	Storage period (days)			SEM	*P-*value		
			3	15	45		D	S	D × S
Corn stalk	ACE	D0	367.49	382.19	321.66	5.29	0.981	0.040	0.603
		D1	368.14	385.99	333.29				
		D3	354.87	362.02	364.79				
		D5	375.11	368.72	344.03				
	Chao1	D0	239.00	253.20	222.87	3.28	0.861	0.183	0.396
		D1	245.43	236.87	213.73				
		D3	236.10	225.03	241.90				
		D5	249.07	236.23	231.23				
	Shannon	D0	2.43	2.43	2.30	0.04	<0.001	0.080	0.191
		D1	1.82	2.03	1.85				
		D3	1.53	1.75	2.08				
		D5	1.64	1.92	2.02				
	Simpson	D0	0.16	0.15	0.18	0.01	0.001	0.015	0.190
		D1	0.34	0.23	0.31				
		D3	0.40	0.32	0.21				
		D5	0.39	0.26	0.23				
*Pennisetum sinese*	ACE	D0	334.05^AB^	368.47^A^	279.72^Bb^	4.26	0.116	0.002	0.012
		D1	336.63	361.45	348.33^a^				
		D3	345.29^Ab^	390.27^A^	309.27^Bb^				
		D5	340.32	355.38	375.00^a^				
	Chao1	D0	142.47	155.40	129.27^b^	2.82	0.155	0.285	0.043
		D1	143.47	151.53	162.80^a^				
		D3	155.70^AB^	174.60^A^	129.83^Bb^				
		D5	156.57	154.23	173.50^a^				
	Shannon	D0	2.02	2.18	1.99	0.03	0.917	0.738	0.630
		D1	2.04	2.07	2.08				
		D3	2.08	2.09	1.89				
		D5	2.01	2.04	2.20				
	Simpson	D0	0.25	0.23	0.24	0.01	0.958	0.553	0.936
		D1	0.22	0.24	0.25				
		D3	0.21	0.25	0.27				
		D5	0.22	0.25	0.23				

Means within the same row (A–B) or within the same column (a–b) with different superscripts differ significantly from each other (*P* < 0.05).

D0, undiluted; D1, 10^–1^ dilution; D3, 10^–3^ dilution; D5, 10^–5^ dilution; D, dilution gradient; S, storage period; D × S, interaction between dilution gradient and storage period; SEM, standard error of the mean.

**FIGURE 1:**
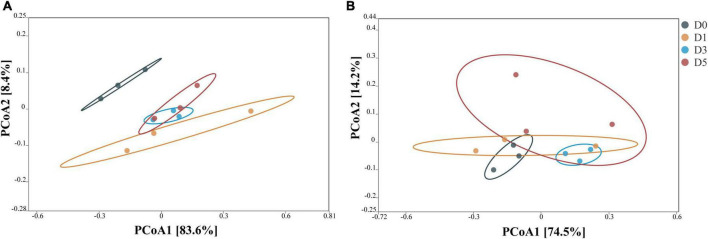
Principal coordinate analysis (PCoA) of the bacterial community from corn stalk silage **(A)** and *Pennisetum sinese* silage **(B)** after 45 days of ensiling. D0, undiluted; D1, 10^– 1^ dilution; D3, 10^– 3^ dilution; D5, 10^– 5^ dilution.

The principal coordinate analysis (PCoA) of the bacterial community after 45 days of ensiling is shown in Figure 1. In corn stalk silage, bacterial diversity was similar between the D3 and D5 treatments, although the bacterial diversity of D0 different significantly from those of the other treatments. In *P. sinese* silage, both the D3 and D0 treatments showed distinct clustering, while the D1 and D5 treatments exhibited some overlap. These results indicate that the dilution and enrichment of epiphytic microflora had an impact on the bacterial community of silage.

**FIGURE 2:**
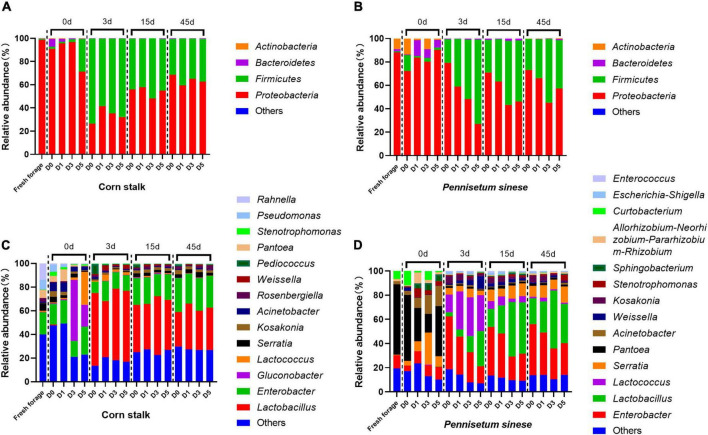
Bacterial community structure and relative abundance, at the phylum [corn stalk, **(A)**; *Pennisetum sinese*, **(B)**] and genus [corn stalk, **(C)**; *P. sinese*, **(D)**] levels, of raw forage and silage. D0, undiluted; D1, 10^– 1^ dilution; D3, 10^– 3^ dilution; D5, 10^– 5^ dilution.

The bacterial community composition and relative abundance, at the genus and phylum levels, of both forage and silage are shown in Figure 2. Proteobacteria was the most common phylum in both fresh corn stalk (98.50%) and fresh *P. sinese* (87.99%). In fresh corn stalk, the dominant genera were *Enterobacter* (18.60%), *Rahnella* (13.93%), *Pseudomonas* (8.29%), *Pantoea* (6.88%), and *Serratia* (6.36%). In fresh *P. sinese*, the dominant genera were *Pantoea* (58.18%), *Enterobacter* (10.68%), and *Curtobacterium* (6.69%), of which only *Curtobacterium* belongs to the phylum Actinobacteria. After enrichment of the epiphytic microflora of corn stalk, the D5 treatment exhibited a high abundance of *Lactococcus* (27.80%), and *Gluconobacter* was enriched in both the D3 (51.49%) and D5 (18.47%) treatments. After enrichment of the epiphytic microflora of *P. sinese*, the D0 treatment exhibited a high abundance of the phylum Firmicutes, primarily represented by the genus *Weissella*. In the D1, D3, and D5 treatments, the abundance of *Pantoea* decreased, while the abundances of *Serratia*, *Acinetobacter*, and *Sphingobacterium* increased. After the initiation of anaerobic fermentation, the abundance of *Gluconobacter* decreased in the D3 and D5 treatments in corn stalk silage, and the abundances of *Pantoea*, *Curtobacterium*, *Acinetobacter*, and *Sphingobacterium* decreased in *P. sinese* silage. In contrast, the abundance of Firmicutes increased substantially after ensiling. In corn stalk silage, this phylum was primarily represented by *Lactobacillus*, while in *P. sinese* silage, this phylum was represented by both *Lactobacillus* and *Lactococcus*. In addition, both corn stalk and *P. sinese* silages contained a high abundance of *Enterobacter*.

**FIGURE 3:**
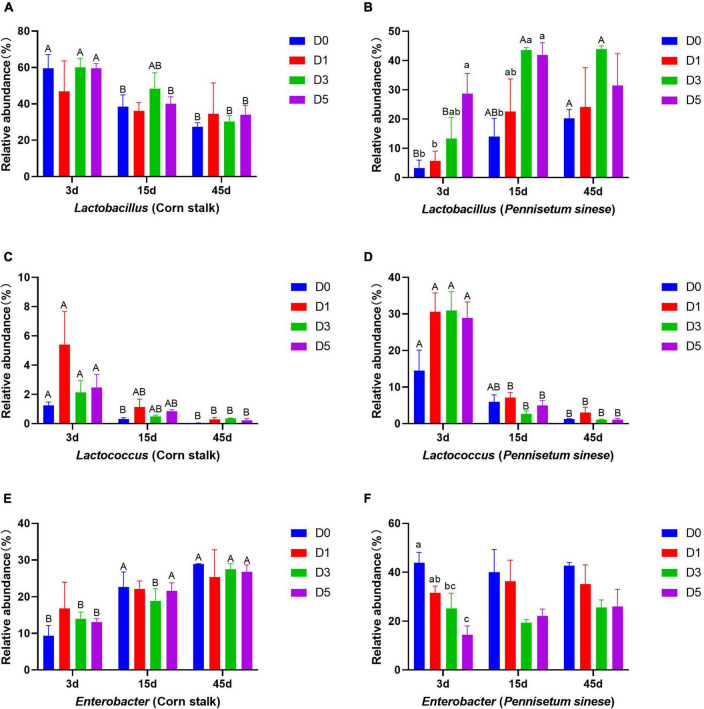
Changes in the relative abundances of dominant bacterial genera, including *Lactobacillus* [corn stalk, **(A)**; *Pennisetum sinese*, **(B)**], *Lactococcus* [corn stalk, **(C)**; *P. sinese*, **(D)**], and *Enterobacter* [corn stalk, **(E)**; *P. sinese*, **(F)**], during ensiling. D0, undiluted; D1, 10^– 1^ dilution; D3, 10^– 3^ dilution; D5, 10^– 5^ dilution. Means within the same storage period (a, b, c) or within the same dilution gradient (A, B) with different superscripts differ significantly from each other (*p* < 0.05). Error bars indicate standard error of means.

Changes in the relative abundances of dominant bacterial genera are shown in Figure 3. In corn stalk silage, the abundance of *Lactobacillus* decreased significantly with storage time, while the abundance of *Enterobacter* increased significantly (*P* < 0.05). In *P. sinese* silage, the abundance of *Lactobacillus* tended to increase in the later stages of anaerobic fermentation. The abundance of *Lactococcus* decreased significantly (*P* < 0.05) with time in both silages.

## Discussion

The distribution of dominant genera has been found to be related to the type of forage (Li et al., 2019). As expected, we found that the microflora attached to corn stalk and *P. sinese* differed significantly. Additionally, the dilution and enrichment treatments did cause reconstitution of the epiphytic microflora of the forages. The abundance of LAB in the pre-incubated and microbially enriched forage juices did not increase as overwhelmingly as in the traditional pre-fermented forage juice during the enrichment process, and the dominant bacterial phylum remained *Proteobacteria*, which is likely related to the aerobic incubation environment. Similar to the general silage fermentation process, the abundance of undesirable microorganisms belonging to Proteobacteria greatly decreased after anaerobic fermentation. Proteobacteria and Firmicutes are frequently found to be the most dominant phyla in silage (Wu et al., 2020; Chen et al., 2021). The transition from Proteobacteria to Firmicutes is common during the switch from an aerobic to an anaerobic environment. Anaerobic and low pH conditions facilitate the growth of Firmicutes, which have acidogenic, acid hydrolytic, and enzymatic hydrolytic functions (Ali et al., 2020; Chen et al., 2020).

The relatively high abundance of a small number of dominant bacteria in the later stages of ensiling may have led to an overall decrease in bacterial community richness. Additionally, the dilution and enrichment treatments may have reduced the competitiveness and abundance of certain bacteria, leading to a decrease in bacterial diversity and alterations to the bacterial community structure. Similar results have been reported by others, notably in Philippot et al. (2013) soil study, in which the diversity metrics indicated a decrease in diversity in the 10^–3^ and 10^–5^ dilution treatments compared with the undiluted one.

The D3 treatment in corn stalk silage and the D3 and D5 treatments in *P. sinese* silage exhibited decreased levels of hemicellulose, indicating effective fiber degradation. Correspondingly, there was a tendency toward higher *Lactobacillus* abundance in all the above treatments at day 15, thus contributing to lower pH. Hemicellulose is less stable than cellulose, and is less resistant to acid and alkali hydrolysis, and organic acids generated during fermentation often lead to the partial hydrolysis of hemicellulose (Ning, 2016). Hemicellulose exhibits extensive decomposition at pH 4.0, and produces significantly more reducing sugars at pH 4.0 than at pH 5.0 or 6.0 (Dewar et al., 1963). These results confirm that the reconstitution of the epiphytic microflora caused by the dilution and enrichment pretreatments led to alterations in both the microbial metabolites and chemical composition of the silages.

Generally, enterobacteria are the second most abundant bacterial group found in silage. Enterobacteria proliferated rapidly at the beginning stages of fermentation, competing with LAB for substrate and primarily producing acetic acid. Silage with pH below 4.5 is effective at inhibiting the growth of enterobacteria (Rooke et al., 1990; Pahlow et al., 2003). *Lactococcus* often grow vigorously at the beginning of ensiling and initiate lactic acid fermentation, thus stimulating the subsequent dominance of *Lactobacillus* (Ni et al., 2018). The low abundance of *Lactococcus* may have been responsible for the decreased *Lactobacillus* abundance at later fermentation stages in corn stalk silage. Additionally, although there was an increase in the *Lactobacillus* abundance in *P. sinese* silage, it was not enough to inhibit *Enterobacter*. Previous studies have found that corn stalk silage with a high moisture content is susceptible to a high abundance of *Enterobacter* (Xu et al., 2018; He et al., 2020). Contrary to our expectations, pre-treatment with fermented forage juice did not result in LAB dominance in treated silages. This may have been due to the strong inhibition of LAB by aerobic bacteria during the enrichment process, thus affecting subsequent anaerobic fermentation. The LAB generally grow satisfactorily in the absence of oxygen, and in its presence some are inhibited partially or completely. Therefore, it is rather common to conclude that the normal growth metabolism of LAB is anaerobic and that metabolism in the presence of oxygen is somewhat aberrant (Condon, 1987). In contrast, aerobic microorganisms can proliferate better in the air. A similar phenomenon can be observed in the aerobic feed-out phase of silage, where aerobic microorganisms get rid of the inhibitory effect of anaerobic condition and proliferate rapidly after exposure to the air, weakening the dominance of LAB and causing spoilage of the silage (Wilkinson and Davies, 2013).

The presence of a high abundance of *Enterobacter* resulted in high levels of acetic acid in both corn stalk and *P. sinese* silages. High levels of acetic acid tend to reduce the intake of silage by livestock. However, an acetic acid content above 5% (DM) can increase the aerobic stability of silage by more than 100 h (Danner et al., 2003). Therefore, a high concentration of acetic acid is beneficial for corn stalk silage, which is extremely susceptible to aerobic spoilage (da Silva et al., 2018). In the present study, only the D3 treatment of corn stalk silage exhibited pH below 4.2, suggestive of high quality fermentation (McDonald et al., 1991). The D3 treatment also exhibited a relatively high content of acetic acid. Although the acetic acid was most likely not produced from heterofermentative LAB, its improvement of aerobic stability may be beneficial nonetheless.

From a bioenergy production standpoint, acetic acid can be used as a substrate for hydrogen production (Khanal, 2009a). Acetic acid and hydrogen are also the primary substrates for the production of methane (Khanal, 2009b). Although acetic acid can be further utilized, acetic acid fermentation-based silage still results in higher biomethane losses compared with lactic acid fermentation-based silage (Sun et al., 2021). Here, although we did not see butyric acid fermentation dominated by clostridia, which can result in the greatest biomethane losses, corn stalk silage did not exhibit superior potential as a bioenergy source compared to *P. sinese* silage, due to *Enterobacter* proliferation and acetic acid fermentation.

## Conclusion

Both corn stalk and *P. sinese* silages exhibited a high abundance of *Enterobacter* during ensiling, resulting in high levels of acetic acid and potentially improved aerobic stability. The D3 treatment of corn stalk silage resulted in the highest fermentation quality. From a bioenergy standpoint, the treatments did not produce effective lactic acid fermentation, suggesting that dilution and enrichment of the epiphytic microflora of corn stalk or *P. sinese* is not an ideal strategy for minimizing biomethane loss. However, the reconstitution of the epiphytic bacterial communities on the forages was found to alter the microbial metabolites and chemical composition of the silage. These results provide a new perspective on the production of pre-fermented silage inoculant.

## Data availability statement

The data presented in this study are deposited in the NCBI Sequence Read Archive (SRA) repository, accession number: PRJNA892779.

## Author contributions

PL and CC designed the study. YX wrote and revised the manuscript. ED, YY, and WW carried out the data analysis. YY, WW, XH, HS, YZ, QC, and CW performed the experiments. All authors reviewed and approved the final manuscript.
